# Giant splenic artery aneurysm: A case report

**DOI:** 10.1016/j.ijscr.2025.112126

**Published:** 2025-10-27

**Authors:** Visagan Srimurugathas, Vinojan Satchithanantham, Mathievanan Sivalingam, Sriluxayini Mayorathan, Mayuran Murugavel, Umashankar Kanthasamy

**Affiliations:** a Post Graduate Institute of Medicine University of Colombo, Sri Lanka; b Faculty of Medicine Jaffna, Sri Lanka; c Teaching Hospital Jaffna, Sri Lanka

**Keywords:** Giant splenic artery aneurysm, Visceral artery aneurysm, Endovascular therapy, case report

## Abstract

**Introduction:**

Giant splenic artery aneurysm is a rare entity but can be potentially life threatening if ruptured.

**Presentation of case:**

This case report describes a 77-year-old male with a giant splenic artery aneurysm (12x11x9.7 cm), presenting with persistent left hypochondrial discomfort. The patient underwent open aneurysmectomy and splenectomy. The patient's recovery was uneventful, highlighting surgical management's importance in complex cases.

**Discussion:**

The treatment for splenic artery aneurysm can be open surgical or endovascular based on the size, morphology, the available expertise and the fitness of the patient. Endovascular therapy is less invasive and low morbidity with high success rate.

**Conclusion:**

Open surgical repair with or without splenectomy is a successful treatment modality for giant splenic artery aneurysm.

## Highlights

• Giant splenic artery aneurysm is a rare entity but a potentially life threatening one if ruptures
• This case report highlights a symptomatic giant splenic artery aneurysm
• The patient underwent open aneurysmectomy with splenectomy
• Complex large giant splenic artery aneurysms can be successfully managed with open surgery

## 1 Introduction

Splenic artery aneurysm (SAA) is defined as an abnormal dilatation of the splenic artery more than 1 cm in diameter. The mean diameter of these lesions was 3.1 cm, whereas the maximum diameter did not exceed 5 cm [1]. True giant splenic artery aneurysms (GSAAs) >5 cm are uncommon [1].

Splenic artery aneurysms are the third most common intra-abdominal aneurysms, following aortic and iliac aneurysms, and account for approximately 60 % of visceral artery aneurysms [2]. They are relatively uncommon with estimated prevalence of 0.8 % [3]. They are clinically significant due to their potential for rupture. This case report has been reported in line with the SCARE checklist [4].

## 2 Presentation of case

A 77-year-old male presented to us with a complaint of left hypochondrial discomfort that had persisted for four days. He denied experiencing any associated symptoms such as vomiting, nausea, fever, or weight loss. His bowel habits were normal, and he reported no changes in urinary habits. His medical history was negative for any previous episodes of pancreatitis, and there were no signs or symptoms of infection. He is not on any drugs and had no known allergies for drugs or food.

On admission, his vital signs were within normal limits. On abdominal examination, a pulsatile mass was palpable in the left hypochondrium, and a bruit was audible over the mass.

**Fig. 1:**
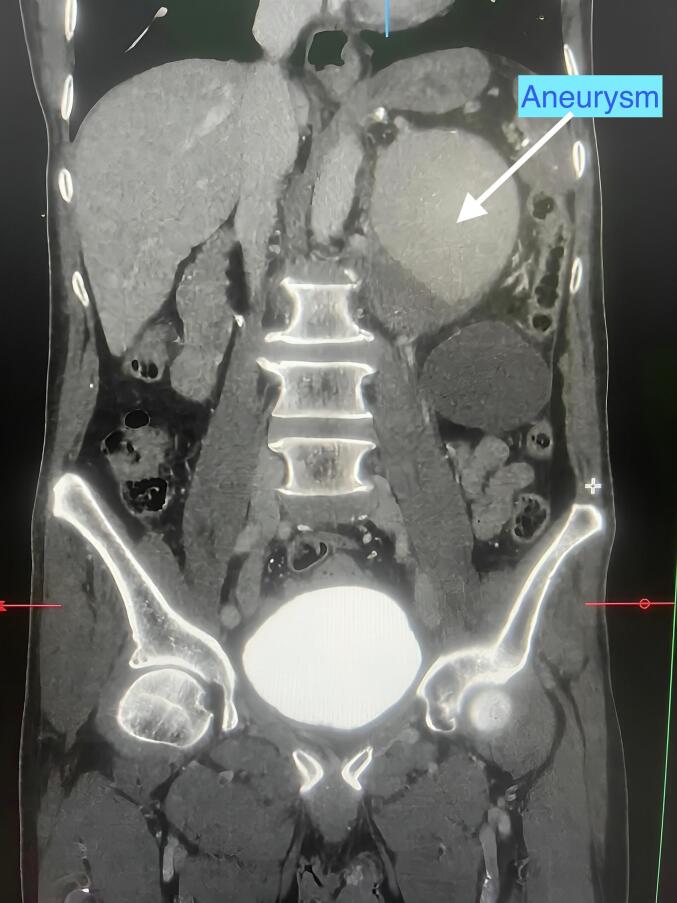
Contrast Enhanced CT of Abdomen showing Giant Splenic Artery Aneurysm

Initial imaging with ultrasound (USS) of the abdomen revealed a cystic lesion in the peripancreatic area. Further evaluation with a contrast-enhanced computed tomography (CECT) of the abdomen confirmed a large splenic artery aneurysm measuring 12 × 11 × 9.7 cm (See Fig. 1). The aneurysm exhibited peripheral thrombus and calcifications, confirming the diagnosis of a giant splenic artery aneurysm.

**Fig. 2:**
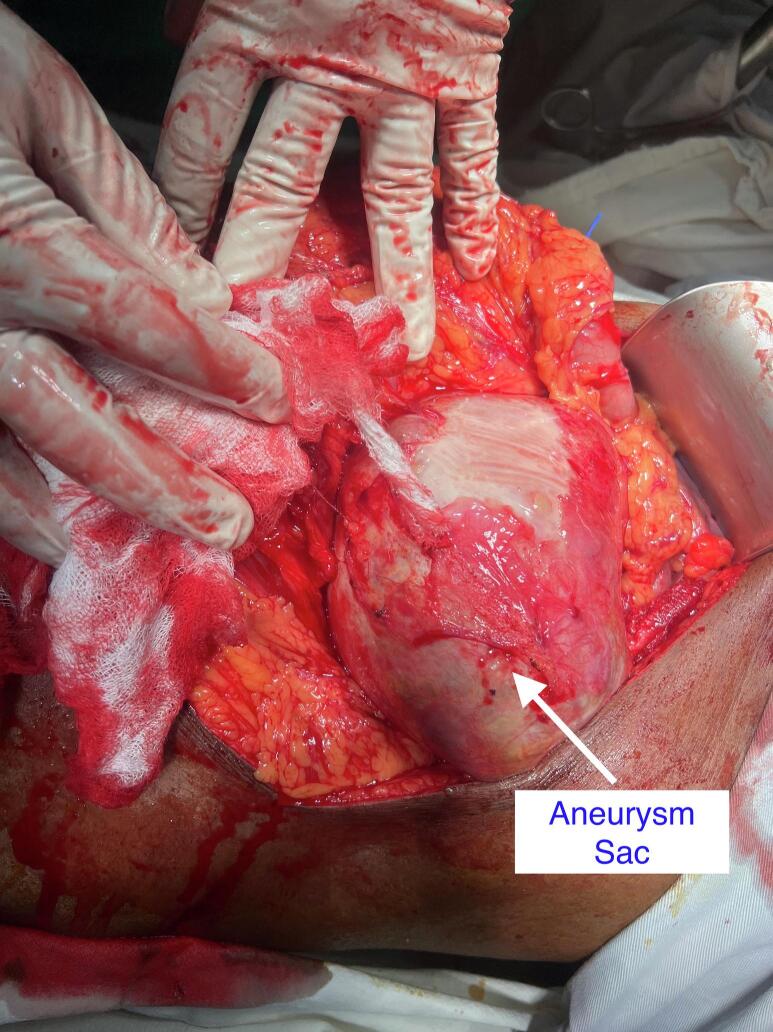
Intraoperative picture showing GSSA.

**Fig. 3:**
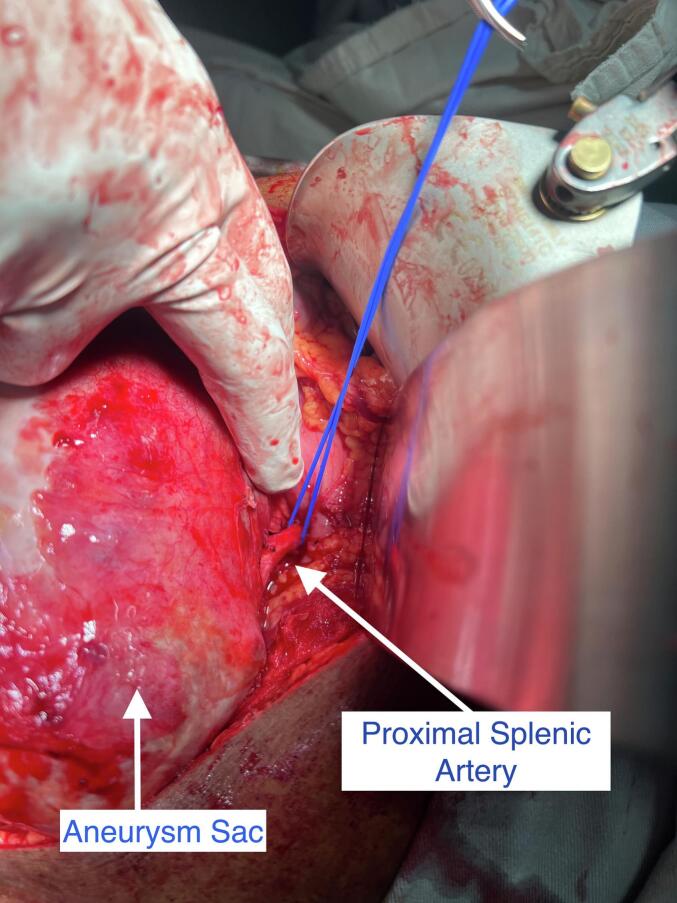
Intraoperative picture showing ligation of splenic artery

We decided to proceed with the open surgery as appropriate coils were not available at that time and considering the large size of the aneurysm causing pressure symptoms. Roof top incision was made and the splenic artery was ligated in the lesser sac (See Fig. 2, Fig. 3). Initially we attempted to excise the whole aneurysm sac, as the posterior wall of the sac was adhered to pancreas it was decided to leave the wall adhered to pancreas. Rest of the aneurysm was excised. At the end of the operation the spleen looked ischemic, and splenectomy was done. The patient was discharged after a week of surgery. He was reviewed in one week and one month later and his postoperative period was unventful.

## 3 Discussion

Abbas MA, Stone WM, Fowl RJ, et al. stated that splenic artery aneurysm is four times more common in women but approximately three times more likely to rupture in men [5] with mortality rates as high as 25–70 % in untreated cases. Though rare, SAAs have been reported more frequently in recent years, possibly due to advancements in imaging technologies and the incidental discovery of these aneurysms during evaluations for unrelated conditions. Giant pseudoaneurysms (more than 5 cm in size) of splenic artery are also extremely rare and about 20 cases have been reported, largest being 19 cm as reported by Goldberg et al. in 2010 [6]. The mean age at presentation of GSAA was 56 years, which is younger than the mean age of 61 years reported for common SAA [7].

Splenic artery aneurysms can be categorized as true or pseudo aneurysm based on involvement of layers or based on size or shape. Our case is a true aneurysm. Most of the aneurysms develop in the trunk of the artery (75 % in distal one third and 20 % in middle one third) and they are single and saccular in shape [8,9].

The etiology of SAAs remains varied, with risk factors including atherosclerosis, hypertension [10], portal hypertension [10], and pregnancy [11]. Conditions such as cirrhosis [10] and liver transplant [12] have also been associated with an increased incidence of these aneurysms due to altered hemodynamics in the splenic artery. But multiple giant splenic artery aneurysm causing portal hypertension also reported in one case [13].

The pathogenesis of splenic artery aneurysm is explained as local haemodynamic changes and hormonal factors causing damage to vascular vessel components such as impaired elastin formation, degeneration of internal elastic lamina and medial degeneration. Risk factors for rupture of the aneurysms include pregnancy [11] development of symptoms, expanding aneurysms, a diameter greater than 2 cm, portal hypertension [10], portocaval shunt [10] and liver transplantation [12].

Hytham K. S. Hamid et al. mentioned that almost three-fourths of GSAAs were symptomatic at the time of the initial diagnosis, and most, particularly distal lesions, had symptoms directly related to aneurysm [7]. The rupture rate for GSAA was 34.8 % compared with only 2 % to 10 % for common SAA [7]. Symptoms can be due to mass effect, rupture or secondary to portal hypertension. Abdominal pain is found as the most common presenting symptom [7]. Pain is in the epigastrium or left upper quadrant.

Sudden onset sharp abdominal pain in the epigastrium or more often left upper quadrant, left shoulder tip pain(Kehr's Sign) and hemodynamic instability suggest rupture which is reported to occur in 2–10 % of patients as the initial presentation [6]. Patients may also present with erosion of aneurysm into adjacent viscous may cause gastrointestinal hemorrhage or into splenic vein can cause arteriovenous fistula which may present with portal hypertension or mesenteric steal syndrome due to high flow. Spontaneous Rupture is the most serious complication of SAA with an overall mortality rate of 25 % [14]. Dave et al. reported 3 cases of rupture in 9 patients (33 %) in 2 of the patients who had ruptured, diameter was larger than 3cm [15]. In the largest series, among 19 patients with “small” aneurysms (mean diameter 1.4 cm) not operated on, no rupture or other complications occurred [16]. Apart from rupture GSSA can cause splenic infarction and rarely it can compress biliary tract to cause obstructive jaundice [6].

Ultrasound, CT, MRI, MRA, and endoscopic ultrasound are all imaging modalities commonly used to initially diagnose an asymptomatic aneurysm. Ultrasound (US) is non-invasive, cost-effective, and radiation-free imaging method, making it particularly suitable for use during pregnancy but accuracy is low as it can suggest only cystic lesions in the region in our case. CT scans and MRI offer the benefit of three-dimensional cross-sectional imaging, but each has its limitations. Once initial suspicion or diagnosis is made, digital subtraction angiography (DSA) is highly valued for its spatial resolution, allowing for precise identification of the aneurysm's location, assessment of collateral circulation, localization of bleeding sources, and confirmation or exclusion of additional visceral aneurysms. However, DSA is invasive, requiring arterial puncture and carrying certain procedural risks. In contrast, multi-detector CT (MDCT) does not require arterial puncture and, with its three-dimensional rendering capabilities, enhances the assessment of splenic artery aneurysms (SAAs).

Splenic artery aneurysm can be managed conservatively with regular imaging follow up or can be managed with intervention. For the aneurysms exceeding 2 cm there is general agreement that elective surgical treatment is indicated, although further studies are necessary to find out the real incidence of rupture in this condition [17]. Other indications for intervention include symptomatic patients, women of childbearing years, pregnancy, and cirrhotic patients planning to undergo orthotopic liver transplantation or porto venous shunting procedures. The risk-benefit ratio should be carefully considered for patients with asymptomatic lesions smaller than 2 cm. In this case, the lesion measured 12 cm, prompting the decision to proceed with surgical intervention.

Endovascular treatment options, such as transcatheter embolization, percutaneous injection, and endovascular stent grafts. Fusiform true aneurysms can be treated with a stent graft, while tortuous, saccular aneurysms are treated with aneurysmal coiling techniques [18]. Endovascular procedures are considered first choice for SSA which offer low post operative morbidity and mortality and early recovery and minimal invasiveness. Technical success rate was 96 % (47 patients out of total 49) in an analysis done by Ryan O. Lakin et al. [19] But these are limited by tortuosity of arteries small artery dimensions and location of the lesion. Open surgery has advantage of long term outcome tortuos artery and proximal SSA and it is also indicated when endovascular treatment fails as in our case. Intraoperative precautions included early vascular control to prevent catastrophic hemorrhage, careful handling of the pancreas to avoid postoperative pancreatitis or fistula, and preservation of splenic perfusion where feasible.

A recent literature review by Hse Juinn Lim revealed there is no significant difference in mortality between three different interventional treatment modalities [20]. The suitable size of coils were not available for endovascular treatment in our hospital settings. Therefore once the endovascular treatment failed decision to go for surgery was taken. Surgical procedure contains ligation of the aneurysm, aneurysmectomy with or without splenectomy. SAAs that are proximally located, elongated, and tortuous are well-suited for aneurysmectomy with end-to-end reconstruction without requiring splenectomy [21]. However, for lesions arising in the distal two-thirds of the splenic artery, splenectomy may be necessary [21].

Although splenic preservation was the preferred approach, intraoperative assessment revealed a dusky spleen with patchy infarction, absent capillary bleeding, and loss of arterial pulsation, indicating irreversible ischemia in our case. In view of these findings, splenectomy was performed, as preservation was deemed infeasible and unsafe. Raffaele Pulli, M.D., suggested several reasons to avoid splenectomy such as higher susceptibility to infection, increased operation time and surgical stress, greater blood loss during surgery [17], higher risk of post splenectomy sepsis we were not able to preserve it due poor viability of spleen after aneurysmectomy. During the surgery the adequacy of splenic blood supply is tested by observing color of spleen and venous filling. Splenic perfusion persists through collateralization from the short gastric vessels. Aneurysmectomy with end-to-end anastomosis for splenic artery reconstruction is feasible, as these aneurysms are frequently saccular, and the splenic artery is generally tortuous and has excess length [17].

## 4 Conclusion

Giant splenic artery aneurysm can present as pulsatile mass in left upper quadrant of abdomen. Giant splenic artery aneurysms have a high risk of rupture causing high mortality rate and need prompt management to prevent that. The endovascular therapy can be attempted as it is less invasive and low morbidity. Elective surgical repair can be done successfully with good outcomes if the endo vascular options fail or in unavailability.

## Ethical approval

Our Institutional Review Board does not require ethical approval for reporting individual cases. The ethical clearance is not necessary for this study. Written informed consent for publication of the clinical details and accompanying images was obtained from the patient, and confidentiality has been strictly maintained.

## Funding

This research did not receive any specific grant from funding agencies in the public, commercial, or not-for-profit sectors.

## Author contribution

Srimurugathas Visagan: Conceptualization, Writing – Original Draft, Satchithanantham Vinojan: Conceptualization, Supervision, Resources, Sivalingam Mathievanan: Resources, Sriluxayini Mayorathan: Resources, Murugavel Mayuran: Resources, Kanthasami Umashankar: Resources.

## Conflict of interest statement

The authors declare that they have no known competing financial interests or personal relationships that could have appeared to influence the work reported in this paper.

## Guarantor

Srimurugathas Visagan

## Research registration number

Not applicable

## Consent

Informed written consent was obtained from the patient for publication of this case report and accompanying images. A copy of the written consent is available for review by the Editor-in-Chief of this journal on request.
